# Pelvic Inflammatory Disease—What Is True Conservatism in Its Treatment?

**Published:** 1895-04

**Authors:** 


BUFFALO MEDICAL AND SURGICAL JOURNAL
A MONTHLY REVIEW OF MEDICINE AND SURGERY.

*EDITORS:*

THOMAS LOTHROP, M. D. - - WM. WARREN POTTER, M. D.

All communications, whether of a literary or business nature, should be addressed to the managing editor: 284 FRANKLIN STREET, BUFFALO, N. Y.

### PELVIC INFLAMMATORY DISEASE—WHAT IS TRUE CONSERVATISM IN ITS TREATMENT?

A REMARKABLY interesting discussion was held at the last meeting of the American Association of Obstetricians and Gynecologists, in which the history, clinical features, causation, pathology, diagnosis, prognosis and treatment of inflammatory disease of the uterus, its appendages and of the pelvic peritoneum were exhaustively considered. Some eight or nine referees took part in the discussion and these gentlemen were exceedingly well chosen, for they were fitted by experience and training to deal with the subject in a masterful manner.

This discussion was published in the *American Journal of Obstetrics* as well as in the transactions of the association, hence has become the property of the profession and is a most valuable contribution to the literature of gynecology—perhaps the most important one made in this country during the year 1894.

The frequency with which the diseases in question—acute or chronic, primary or secondary—are met, makes the subject one of absorbing interest to every practicing physician whose province is to deal with the pelvic organs of women. It is particularly important that the family doctor should be well versed in the clinical history and diagnosis of these affections in order that he may be prepared, either in person or by appropriate counsel, to make early application of rational and effective treatment. It is, moreover, of paramount importance that he shall not be misled by the dangerous doctrines that have been lately preached under the disguise of the misapplication of the term conservatism.

In reading this discussion we have become particularly impressed with the clearness and forcefulness of the arguments made on that point. There has sprung up, of late, an attempt on the part of a few men, who heretofore have been regarded in a certain sense as leaders, to decry the surgical treatment of these affections and to prove that they can be cured by tentative measures, such as can be applied during a long residence in a private hospital. These, of course, include rest, massage, electricity, diet and almost every known luxury that a proud purse can provide. It is not to be contended that these remedies have not a place in certain well-chosen cases and in appropriate environment; but it cannot be affirmed with any approach to truthfulness that they are to be employed for the cure of pus-laden tubes, cystic ovaries or in cases where the pelvic tissues, through neglect or otherwise, have become matted together until their anatomical outlines are no longer to be mapped out.

The debaters in the discussion above referred to have, in several instances, paid their respects to this dangerous teaching in unmistakable terms, and we believe the effect of their arguments will go far towards checking the false position into which many good men were being betrayed. At all events we have observed that the medical journals throughout the country have ceased to echo these taking, but dangerous, arguments. The principles enunciated by experienced surgeons, who have derived their knowledge from long-suffering effort at the operating table, and who are able to point with satisfaction to a long record of cures are, after all and in the long run, more convincing than the pretty vagaries of the theorist.

When pus has ravaged a woman's pelvis until her organs are no longer anatomically recognizable or physiologically useful, it stands to reason that the best way to cure such a patient is through the prompt application of the best known surgical principles; and this is about the sum and substance of the whole matter. It is, at all events, the conclusion that thinking and observing physicians will reach, after listening to all the arguments and observing the results that the opposing methods bring.

---

THE Kansas medical practice bill was defeated chiefly through the influence of the populists. In the house, according to the *Kansas Medical Journal*, Mr. Winters, a populist member, said: "We Western people can't support your plug-hat doctors. We've got a lot of old women who are better than any of them." And this is the kind of argument that prevails against reform in medical education beyond the Mississippi! Time, however, will set this question right. "Time and I," said Mazarin, "against any other two."

